# Simulation Tools for Fog Computing: A Comparative Analysis

**DOI:** 10.3390/s23073492

**Published:** 2023-03-27

**Authors:** Muhammad Fahimullah, Guillaume Philippe, Shohreh Ahvar, Maria Trocan

**Affiliations:** 1 Institut Supérieur d’Électronique de Paris (ISEP), 75006 Paris, France; guillaume.philippe@eleve.isep.fr (G.P.); maria.trocan@isep.fr (M.T.); 2 Nokia Networks, 91300 Massy, France; shohreh.ahvar@nokia.com

**Keywords:** cloud computing, fog computing, edge computing, simulators, evaluation

## Abstract

Fog Computing (FC) was introduced to offer resources closer to the users. Researchers propose different solutions to make FC mature and use simulators for evaluating their solutions at early stages. In this paper, we compare different FC simulators based on their technical and non-technical characteristics. In addition, a practical comparison is conducted to compare the three main FC simulators based on their performance such as execution time, CPU, and memory usage for running different applications. The analysis can be helpful for researchers to select the appropriate simulator and platform to evaluate their solutions on different use cases. Furthermore, open issues and challenges for FC simulators are discussed that require attention and need to be addressed in the future.

## 1. Introduction

Cloud provides on-demand access to a shared pool of resources, which can be accessed by users over the internet on a pay-as-you-go basis such as storage, computing, and network. Over the past few decades, the demands for cloud resources have reached new peaks with an increase in the number of Internet of Things (IoT) devices and the demands for newer services and applications, thus resulting in huge amounts of data generation [1,2]. This rise in demand for cloud resources resulted in numerous challenges within the cloud paradigm. These challenges encompassed factors such as latency, mobility, and location awareness, among other things [3]. Therefore, with the advancement of technologies, cloud-based resources such as storage, computing, and network have been brought near the user’s end to achieve better security, lower latency, manage mobility and improve scalability through a new paradigm known as Fog Computing (FC) [4,5].

This new paradigm introduced in 2012 distributes the resources all the way from the cloud (centralized position) to fog nodes (decentralized positions) near the vicinity of users [6]. Deploying applications or services and managing resources becomes more complex due to the distributed architecture and heterogeneous resources in FC. Therefore, in order to effectively achieve the characteristics of FC, it is required to carry out more and more research in cloud-fog architecture. However, to know the efficiency of these new methods there is a need to evaluate them. Evaluating the proposed methods in the real environment is practically not feasible as failure in the experiment may not only effect other tests but also the existing applications or services as well [1]. Therefore, the researchers mostly focus on other methods such as testbeds and simulators [7]. The testbed can be costly in scenarios where many devices and communication links are involved [8]. Therefore, using a simulator as a solution can be one of the preferred options to test the proposed solution at the early stage of its development [9].

Several simulators are available, each tool providing a set of functionalities. A simulator, suitable for one type of research work may not be suitable for another, and considering a single simulator for every research with different requirements may result in unnecessary delays. Therefore, the selection of an appropriate simulation tool is still a challenge [1,9,10]. Most of the existing literature focuses on the conceptual comparison of simulators as shown in Table 1. A detailed study of simulators from both conceptual and practical perspectives along with future guidelines is required for solving the simulator selection problem. In this work, we aim to analyze the recent and most popular simulators from a technical and non-technical point of view to know where different simulators stand. Furthermore, to get insight into the performance of simulators, we aim to perform practical comparative analysis under different scenarios with varying complexities to know the simulators’ suitability for different types of applications. The key contributions of our work are summarized below.

- We analyze eight recent and most popular fog simulators and compare them from a technical and non-technical point of view (e.g., their current support for different features along with their future goals).
- The performance of three selected simulators is further compared in a practical way by simulating three different applications along with variations in the complexity of scenarios (number of fog and edge devices) and provides discussion on the suitability of simulators for these applications.
- We provide future guidelines for researchers that need attention and need to be addressed in the future.

The rest of the paper is structured as follows. In Section 2, we discuss the related work on simulation comparisons. In Section 3, we provide an overview of the eight simulators and their technical and non-technical comparisons. Furthermore, in Section 4, we provide a practical comparison of three simulators on three different applications under varying complexities. In Section 5, we provide the challenges/issues that the researchers face during the selection of the simulators and finally, we provide our conclusion and future work in Section 6.

## 2. Related Work

This section provides a comprehensive overview of the existing works on simulator comparison. The summaries of the reviewed works are presented in Table 1. Several studies in the literature can be found that provide conceptual comparisons of simulators such as their technical and non-technical characteristics. Considering the technical comparisons, the study in [12], provides a comparative analysis of cloud, edge, and FC simulators. The work concludes that there is still a requirement for a simulator that enables the simulation of complete and complex fog scenarios. The thesis in [13], provides a comparative analysis of simulators and emulators available for Multi-access Edge Computing (MEC) scenarios. The work highlights that the current simulators or emulators are incapable of simulating or emulating security scenarios. The work in [17], provides a comparative analysis of fog and edge simulators. The work also provides modeling and simulation challenges in the fog and edge paradigm. Furthermore, the work in [18], focuses on Edge Computing (EC) simulators. The work concludes that most of the simulators only focus on time and resource utilization while ignoring the other key qualities related to reliability, performance, and security. The work in [21], provides a comprehensive overview of data science tools for IoT technologies. The findings of the study suggest that the majority of the currently available tools either have limited capacity to deliver the efficient performance or are only offered as trial versions (e.g., Matlab).

Furthermore, considering both technical and non-technical comparisons of simulators. The work in [3], provides a comprehensive survey on FC simulators from the perspective of cost. The work highlighted several types of cost issues in their work. However, most of these cost models are adopted by current simulators. However, still, the monetary cost is an open issue that needs to be addressed. A review of simulation tools for FC is presented in [10]. The study compared 27 FC simulators mainly focusing on their technical characteristics. However, the study does not provide any recommendations for simulators based on the scenarios. Furthermore, it focuses on the simulators mainly built in JAVA. In addition, the study in [15], reviews 18 cloud, 18 fog, and 8 IoT simulators. The study provides in detail technical and non-technical comparisons of simulators. However, the study lacks to provide a practical comparison and future open issues that can be addressed. Furthermore, most of the simulators are deprecated or have updated versions therefore, the analysis of the work may not be very useful for recent simulators. Another study in [16], provides a comprehensive overview of a few simulators and emulators for modeling edge, fog, and cloud environments. However, the survey only provides limited discussions on simulators and emulators and lack to provide their applicability details.

In addition, few studies conducted both conceptual and practical comparisons of simulators. For instance, the work in [1], provides a detailed conceptual and practical comparison of 6 cloud-fog simulators. The practical comparison was conducted based on their resource consumption and execution time. Similarly, another study also provides a performance evaluation of cloud and fog simulators in the cloud-fog environment [14]. The work analyzes four simulators from a technical and non-technical point of view. In addition, three of the mentioned simulators (i.e., iFogSim, YAFS, and myiFogSim) were compared practically on the bases of their features (Energy, cost, network, and execution time). Most of the considered tools in these works are updated and therefore, the performance evaluation may not be suitable for the current versions of the simulators. Another study in [11], provides a comprehensive analysis of FC simulators. The work lacks a non-technical comparison of simulators. In addition, resource management aspects of simulators are not considered in their conceptual comparisons. Furthermore, the work provides a practical analysis of one of the simulators considering its resource usage, delay, latency, and simulation time.

Most of the aforementioned works reviewed can be considered outdated. In addition, they do not take into account most of the important technical and non-technical characteristics of simulators such as release frequencies, future goals of the simulators, and more. Furthermore, the existing works lack to provide important open issues that need consideration. Moreover, most of the existing studies only provide technical and non-technical comparisons, ignoring the practical comparisons of simulators on different types of applications. In this paper, we considered additional characteristics in our comparisons along with practical implementations of three different applications and open issues that need to be addressed in new or updated versions of simulators.

## 3. Cloud-Fog Simulators

There are many available simulators that can simulate the scenarios of cloud-fog computing environments such as YAFS [22], LEAF [23], EdgeCloudSim [24], MobIoTSim [25], SimpleIoTSimulator [26], IBM BlueMix [27], Google IoT Sim [28], iFogSim [29], Cooja [30], FogTorch [31], RECAP [32], EmuFog [33]. Most of the available simulators are similar in their functionalities, programming language, or architecture. Therefore, we limited our study to only eight main simulators. The simulators are analyzed both from theoretical and practical perspectives. In theoretical comparisons, all eight simulators (iFogSim, iFogSim2, FogNetSim++, EdgeCloudSim, FogComputingSim, PureEdgeSim, YAFS, and LEAF) are compared based on their technical and non-technical characteristics, whereas for practical comparison three simulators namely iFogSim, YAFS, and LEAF are compared in terms of their execution time, memory usage, and CPU consumption for simulating different applications under varying complexities.

### 3.1. Simulators Overview

In this section, we provide a brief overview of the different simulators mentioned above along with their technical and non-technical characteristics.

#### 3.1.1. iFogSim and iFogSim2

CloudSim [34] and iFogSim [29] were developed by the Cloud computing and Distributed Systems (CLOUDS) Laboratory, a software research and development group within the School of Computing and Information Systems at the University of Melbourne, Australia.

**Figure 1:**
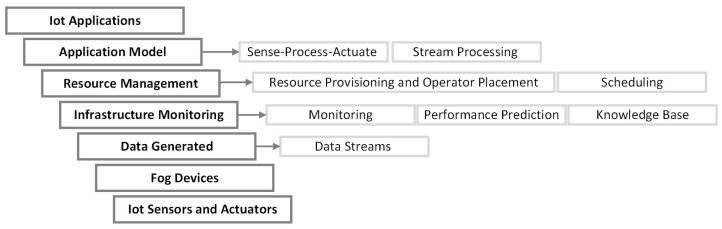
iFogSim Architecture (Adapted from [29]).

The iFogSimToolkit [29], provides a platform for modeling and simulation of resource management techniques in edge, FC, and cloud environments. A newer version of iFogSim [35], was released in 2022, which adds distributed clustering, mobility, and microservices management as new features. Furthermore, it includes new example scenarios to validate and demonstrate their extension for the iFogSim. iFogSim and iFogSim2 are available on GitHub [29,36]. The architecture used by iFogSim is shown in Figure 1.

#### 3.1.2. FogNetSim++

**Figure 2:**
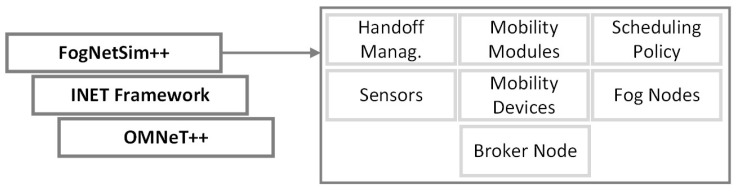
FogNetSim++ Architecture (Adapted from [11]).

The FogNetSim++ [11], is the only simulator in this work based on OMNeT++ [37] and developed in C++. It was introduced in 2018 and there has been no major update since then. The purpose of this simulator was to improve the simulation of the network assuming errors with packets for example. Moreover, it allows researchers to incorporate their own fog node management algorithms, such as scheduling. The simulator is available on GitHub [38] and the architecture used by FogNetSim++ is shown in Figure 2. This simulator has multiple dependencies such as requirements for OMNet++ and INet. In addition, it lacks support for older versions and it is difficult to troubleshoot due to the low number of logs provided. In terms of community support, the project has only eight stars on GitHub, which is the least popular among the other considered simulators.

#### 3.1.3. EdgeCloudSim

**Figure 3:**
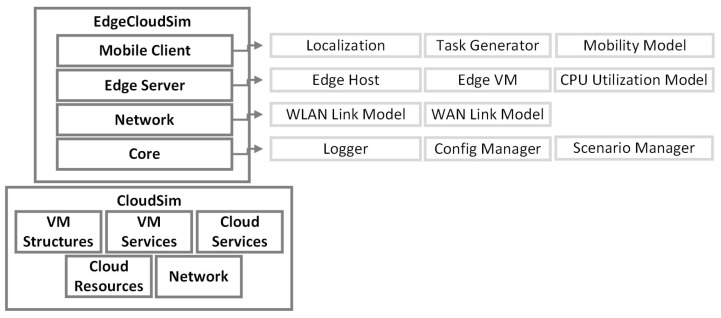
EdgeCloudSim Architecture (Adapted from [24]).

The simulator EdgeCloudSim [24] was introduced in 2018 and is based on CloudSim [34]. However, in order to efficiently utilize it for EC scenarios several functionalities have been added. The architecture used by EdgeCloudSim is shown in Figure 3. EdgeCloudSim provides a modular architecture to provide support for a variety of crucial functions such as network modeling specific to WLAN and WAN, device mobility model, and realistic and tunable load generator. Furthermore, in order to enable users for modeling orchestration actions that arise in EC scenarios, an edge orchestrator module was implemented. EdgeCloudSim is not energy-aware by default [10], but as it implements CloudSim, it can become so. In 2020, they release version 4.0. It includes minor code improvements and some new features requested by the developers (e.g., default constructors). The project is available on GitHub [39]. This is the most popular project of the comparison, with 298 stars and 183 forks on their GitHub repository.

#### 3.1.4. FogComputingSim

The simulator named FogComputingSim [40], was released in 2019. It extends iFogSim [29], and the main features added are the improvement of the user interface and the support of mobility. In the new release of iFogSim, most of these functionalities are added as well. Therefore, this simulator is not very different from iFogSim. The simulator is presented in [40] and it is available on GitHub [41]. It does not provide any installation or documentation manual, but the process is very similar to other Java-based simulators. It requires a CPLEX library for linear programming but the process of launching the scenarios is different from other simulators. Finally, the FogComputingSim console is used to launch the scenarios.

#### 3.1.5. PureEdgeSim

**Figure 4:**
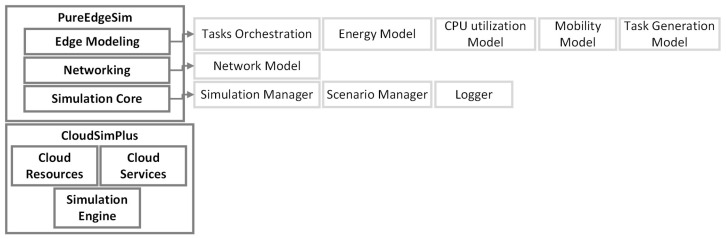
PureEdgeSim Architecture (Adapted from [42]).

Another fog simulator called PureEdgeSim [42] was released in 2019. It extends CloudSim [34] and provides a modular architecture in order to deal with specific parts of the simulation (e.g., network, location). The architecture of PureEdgeSim can be seen in Figure 4. PureEdgeSim allows performance evaluation in terms of resource utilization, delays, network congestion, energy consumption, and task success rate. A load-balancing algorithm was also introduced by leveraging reinforcement learning to adopt to IoT environmental changes.

A new version of the simulator was released in early 2022, which enabled the simulator to support scenarios with tens of thousands of devices and longer simulation times. Furthermore, it provides a measure of the energy consumption of WAN, LAN, MAN, Ethernet, WiFi, and Cellular (5G, 4G, …) networks. The cellular module enables to provide support for the heterogeneous way of communication that is WiFi, 5G, 4G, and Ethernet. However, the simulator does not provide any support for 5G core components architecture. Moreover, PureEdgeSim permits the live visualization of the simulation environment. So when a scenario is executed, the simulator opens a window with live visualization of the simulation. The graph shows network utilization, CPU utilization, tasks success rate, and simulation map. The simulator can be downloaded from the GitHub repository [42]. In terms of its popularity on GitHub, the repository has 85 stars in total.

#### 3.1.6. YAFS

**Figure 5:**
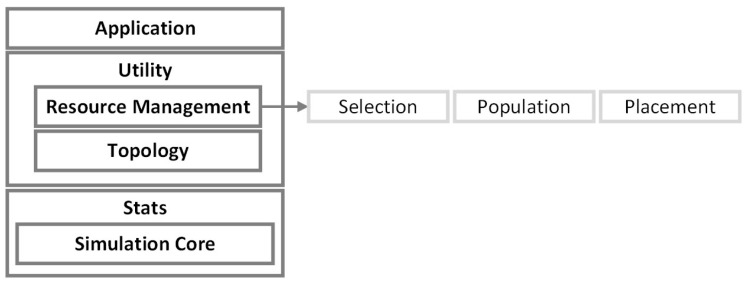
YAFS architecture (Adapted from [22]).

YAFS [22], was first released in 2019. The first version was developed for Python 2.7. YAFS provides dynamic topology (i.e., enabling runtime entities and network link creations), dynamic message creation (i.e., enabling sensors to generate massages at runtime), and users can extend orchestration and placement allocation algorithms. The architecture of YAFS is provided in Figure 5. A new version was released in 2021, allowing support for python 3.6+. They added more example scenarios in the newer version and reduced the third-party libraries’ dependency. However, it is important to remark that some built-in scenarios are not working in the new version due to breaking changes between Python versions. For instance, it can implement scenarios with mobility, but an example scenario (https://github.com/acsicuib/YAFS/blob/master/src/examples/mobileTutorial/main.py, accessed on 16 June 2022) provided is not compatible with python 3.6+. Moreover, some parts of the documentation are not up-to-date. With YAFS, it is easy to generate a plot of the network at any moment. The YAFS repository is available at GitHub [43], it has 55 stars in terms of its popularity on GitHub.

#### 3.1.7. LEAF

**Figure 6:**
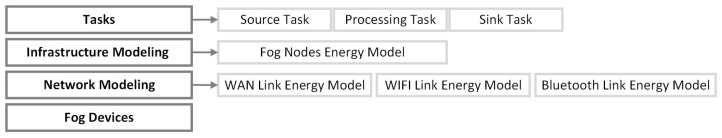
LEAF Architecture (Adapted from [23]).

The most recent simulator of this survey is LEAF and is introduced in 2022 [23]. LEAF can be used for modeling energy-aware scenarios in FC environments. It provides an energy consumption model for edge devices, networks, and data centers. In addition, it provides an energy model for applications running on the infrastructure. The architecture of LEAF can be seen in Figure 6. LEAF is capable of simulating scenarios with thousands of devices and applications at least two times faster than in real-time on normal hardware. LEAF is implemented in both Java and Python. In the future, the authors plan to integrate LEAF with relevant simulators to enable it for simulating and evaluating more realistic scenarios. In addition, functionalities such as location and time-based calculations of energy and carbon emissions will be added. Although LEAF is simple and faster it has a few limitations as well.

The first main limitation is the lack of support for numerous features compared to other simulators. More, it lacks support for bidirectional applications, thus it requires modification in the structure of the application services. Bidirectionally here represents a service that is able to send or receive data. The simulator is accessible via GitHub [44]. Considering its popularity, LEAF has 59 stars on GitHub. This simulator is a recent project, and as they want to introduce new features related to energy consumption, it will be interesting to follow it in the coming years.

### 3.2. Non-Technical Comparison

A simulator having many functionalities does not mean that it is a better simulator. A simple simulator with few functionalities is sometimes better than a simulator with more functionalities. For this reason, we compared the simulators based on their non-technical characteristics such as release dates, stars on GitHub, citation on the articles, frequency of release, response to bugs, and installation manual as shown in Table 2.

Table 2 provides an overview of simulators presented in Section 3. *First Release* refers to the year of the first release of the simulator; *Last Release* corresponds to the year of the latest version release; *Stars* counts the number of stars on the GitHub repository; *Citations* counts the number of citations of the original paper obtained through google scholar; *Paper* references original paper; *GitHub* references GitHub repository of the simulator; *Release Frequency* measures the frequency of updates to the simulator. The *Release Frequency* is measured as moderate when the last update was less than 2 years ago, low when the last update was more than 2 years ago, and deprecated when the repository will no longer be updated; *Response Frequency* measures how often the developers of the simulators respond to an answer when an issue is opened. It is measured in High (response in less than 2 weeks), and Low (no response or response in more than 2 weeks); *Installation* reports if the repository includes installation instructions. Where N/A (Not Applicable) is assigned, no record or details have been found or provided by the simulator.

### 3.3. Technical Comparisons

To better understand the considered simulators, we analyzed the simulators based on two criteria. First, the simulators were compared from a functional point of view as shown in Table 3. Secondly, we considered performance metrics for comparing the simulators as shown in Table 4. Below are the descriptions of each category considered for functional comparisons.

- **Documentation:** Represents whether the simulator is accompanied by documentation, wiki, etc. It is important to note that the quality and completeness of the documentation may vary. Documentation plays an important role in maintaining the simulator by the community.
- **Graphical support:** Represents if the simulator is accompanied by a Graphical User Interface (GUI). In other words, shows if you can build the Fog network architecture using an interface.
- **Migration support:** Depicts whether the simulator has mechanisms for migrating applications from one node to another.
- **Mobility/Location-aware:** Shows whether the simulator supports the motion of IoT devices. This feature is essential for representing real scenarios with moving users.
- **Energy-aware:** Depicts whether the simulator has knowledge of the energy consumption of the architecture and application they are simulating. We decided to separate it into sub-categories: infrastructure, application, network, technology, and carbon emission. These features are crucial to designing less energy-intensive systems.
- **Cost-aware:** Represents whether the simulator has knowledge of the monetary costs involved in FC. This feature is crucial in seeking to optimize deployment, operational, and other costs.
- **Microservices:** Depicts if the simulator supports an orchestration model for microservices deployed across the multi-tier infrastructure.

The performance metrics related to time such as execution, CPU, and network are considered by most of the simulators. Similarly, the performance metrics related to resource consumption such as CPU, memory, and bandwidth are also considered by most of the simulators as shown in Table 4. This can be explained by the common base of several simulators which is CloudSim. On the other hand, the failure metrics (Failed tasks, Waiting, Availability) are less present. In addition, it is important to note that none of the simulators considered or provided carbon emissions while considering energy consumption. Furthermore, most of the simulators provide energy consumption only for infrastructure nodes or networks, ignoring the fact that applications also have an effect on energy consumption performance metrics. Below are the descriptions of each performance metrics considered for simulator comparisons.

- **CPU consumption** gives the CPU usage of the machine running the simulation.
- **Memory consumption** gives the memory usage of the machine running the simulation.
- **Bandwidth consumption** provides the usage of bandwidth during the simulation.
- **Energy consumption** is the energy consumption of each node during the simulation following an energy model. We have decided to distinguish consumption into 5 parts: infrastructure, network (e.g., WAN, LAN), application, technology (e.g., Wifi, Cellular), and carbon emission.
- **Deployment cost** gives the total cost of the simulation following a cost model.
- **Latency** giving the total latency of the simulation.
- **Execution time** is the total execution time of the simulation.
- **CPU time** gives the total CPU time of the machine running the simulation.
- **Network time** is the total time of network usage.
- **Migration time** is the total time passed on migration during the simulation.
- **Failed tasks** is the number of failed tasks during the simulation.
- **Waiting time** is the total time waiting during the simulation.
- **Link availability** provides the total availability of each link.
- **Node availability** provides the total availability of each node.

### 3.4. Summary and Discussion

The technical and non-technical assessment of simulators provides insight into the simulators’ popularity, future directions, and support for different features. In terms of popularity, iFogSim, EdgeCloudSim, and YAFS are the most popular according to their citation count and the GitHub repository usage. Considering features, iFogSim2 and YAFS support the most number of features. However, iFogSim2 supports the important feature of migration (i.e, service) and provides graphical support while YAFS does not. In addition, YAFS provides support for a diverse set of topologies, whereas, iFogSim and LEAF are only restricted to tree topologies. Considering the language support, most of the fog simulators are implemented in JAVA and are based on cloudSim, such as iFogSim, EdgeCloudSim, or PureEdgeSim, and only YAFS and LEAF are developed in python. Furthermore, we observed that most of the simulators’ energy models do not take into consideration the heterogeneity of energy supplies of fog devices (i.e., some fog devices may be running on limited batteries while others may have rich energy resources). In addition, most of the existing methods provide energy models for infrastructure, whereas, energy models of application placed on these fog devices are equally important and are highly neglected by most of the simulators apart from LEAF. Considering the cost models provided by simulators, the pricing of different heterogeneous resources at different levels of FC and their energy supply are highly neglected by most of the simulators as well. From an architectural perspective, all the simulators are capable of modeling cloud-fog continuum scenarios. However, all of them differ from architectural perspectives. For instance, only a few simulators (iFogSim and YAFS) have support for resource management.

## 4. Practical Comparisons

In this section, we performed practical comparisons of three simulators namely: iFogSim, YAFS, and LEAF. The selection of these simulators was based on their latest version of the release and their strong acceptance in the community (GitHub stars, citations, release, and response frequency) along with their support for a rich number of features. For our practical comparison, we considered resource consumption (memory and CPU) and execution time metrics to test the performance of the simulators in implementing different scenarios.

### 4.1. Simulation Framework

The experiments are performed on a computer with the operating system macOS Catalina version 10.15.7. The processor is 2.3 GHz Intel Core i5 2 core and the memory is 8 Go 2133 MHz LPDDR3. The versions of the programming languages used are Python 3.7.12 for YAFS, Python 3.10.0 for LEAF, Oracle Open JDK 18.0.1 for PureEdgeSim, and Java version 1.8.0.

### 4.2. Applications

**Figure 7:**
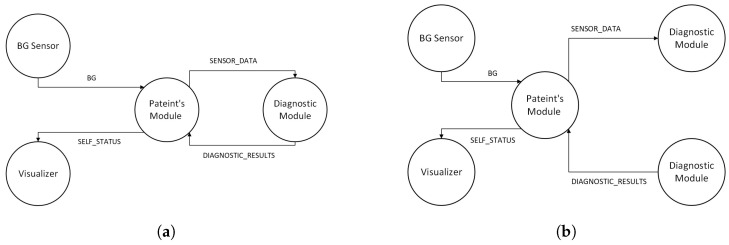
eHealth Application Architecture. (**a**) eHealth Application Architecture used for iFogSim and YAFS. (**b**) eHealth Application Architecture used for LEAF.

**Figure 8:**
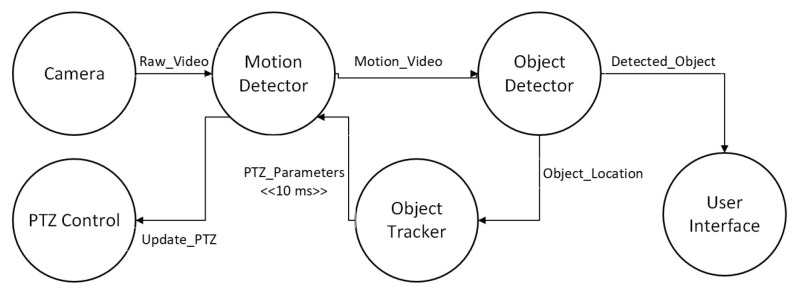
DCNS Application Architecture.

**Figure 9:**
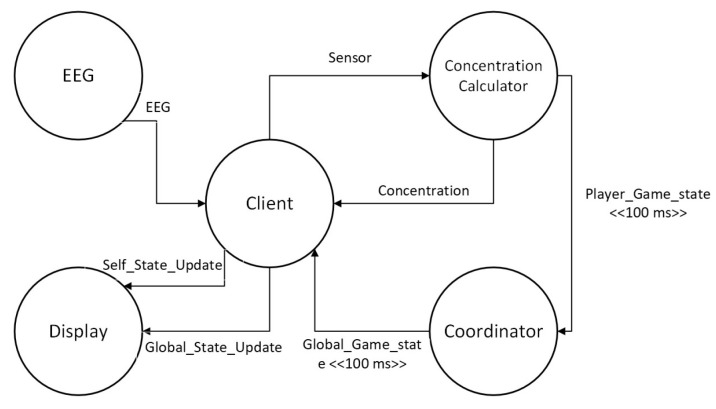
VRGame Application Architecture.

For our experiments, we considered three different types of applications similar to the survey in [1]. The first application we considered is a healthcare application (eHealth) [1]. The patient’s modules collect data from the sensor attached to the patient. The data can be processed by the same module or forwarded to the diagnostic module for further processing. The diagnostic module after processing the data sends back the diagnostic results to the patient module and is visualized as shown in Figure 7a. However, due to a lack of support for bidirectionally in the LEAF, we modified the eHealth application for the LEAF as shown in Figure 7b. The second application we considered is a video surveillance application (DCNS) [1,29]. The DCNS has six modules and the flow of events between these modules can be seen in Figure 8. The third application we considered is a latency-sensitive game (VRGame) [1,29]. It consists of five modules such as EEG, client, concentration calculator, coordinator, and display as shown in Figure 9.

### 4.3. Infrastructure

To evaluate the efficiency of simulators’ performance, the complexity of scenarios is important to take in to account by varying both the resource-constrained fog nodes and IoT devices as well. In this work, we considered different scenarios with varying complexities. The varying number of fog nodes considered for our experiments are 4, 12, and 20, and the number of devices under a single fog node is varied as 4, 8, and 12. We used three layered cloud/fog architecture for our experiments namely cloud layer, fog layer, and IoT layer. Each layer is equipped with resources as shown in Table 5.

### 4.4. Results

All the above scenarios were implemented using iFogSim, YAFS, and LEAF to analyze how well they perform in terms of simulation time (i.e., time to complete the scenario), CPU consumption (i.e., percentage of CPU consumed during the simulation), and memory consumption (i.e., amount of memory consumed during the simulation). The code and the results are available on GitHub [45]. Simulation time is considered in seconds, CPU consumption is considered in percentage and memory consumption is calculated in MB. The results are grouped according to the simulator for each of the scenarios. We performed 10 simulations and recorded the simulation time, CPU, and memory consumption for each experimental setup. For simulation time we used a bar plot to show the average completion time of all the experiments and a box plot to present both the CPU and the memory consumption of all the experiments.

**Figure 10:**
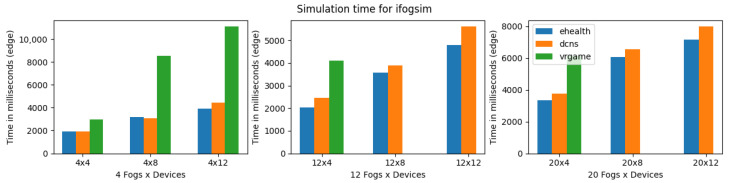
Execution time for iFogSim.

**Figure 11:**
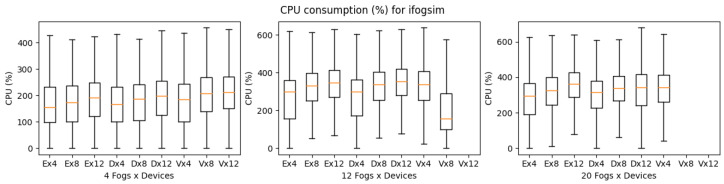
CPU consumption for iFogSim during simulation (E: eHealth; D: DCNS; V: VRGame).

**Figure 12:**
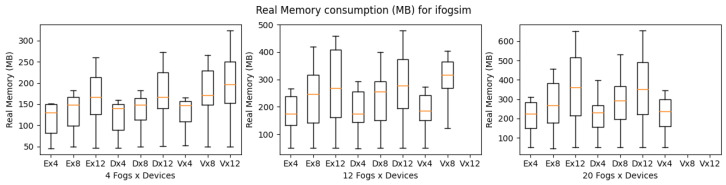
Real memory consumption for iFogSim during simulation (E: eHealth; D: DCNS; V: VRGame).

Figure 10, shows the execution time of iFogSim for eHealth, DCNS, and VRgame. The eHealth and DCNS scenarios simulation times are almost similar in all the experimental setups (4, 12, and 20 fog nodes) with a little advantage to the eHealth application due to its lightweight and fewer services. However, the VRgame application simulation time raises a lot with the increase in complexity of the scenario (increase in the number of fog nodes and edge devices). We consider the CPU and memory consumption of simulators for simulating all three applications. A similar increasing trend in CPU and memory consumption has been observed, as shown in Figure 11 and Figure 12. The most suitable application that performs well in iFogsim is the eHealth application; VRgame performs worst due to scenario complexity increase (fog nodes are 12 and 20), and iFogSim simulator fails to provide the execution time, CPU and memory consumption as can be seen in Figure 10, Figure 11 and Figure 12.

**Figure 13:**
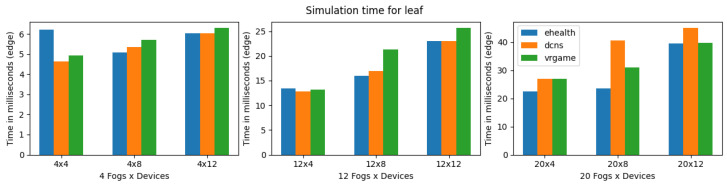
Execution time for LEAF.

**Figure 14:**
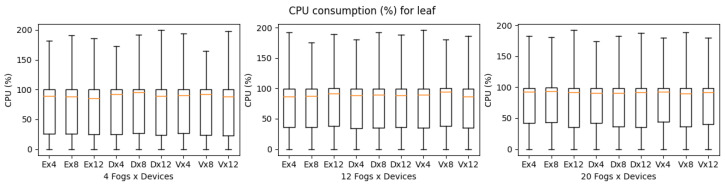
CPU consumption for LEAF during simulation (E: eHealth; D: DCNS; V: VRGame).

**Figure 15:**
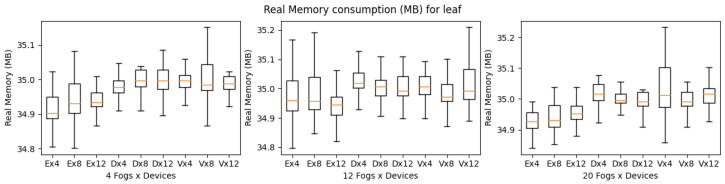
Real memory consumption for LEAF during simulation (E: eHealth; D: DCNS; V: VRGame).

The results of execution time, CPU, and memory consumption are a bit complicated in the case of the LEAF simulator. Compared to iFogsim, LEAF was able to simulate all the application scenarios (eHealth, DCNS and VRgame), regardless of the increase in complexity of the scenarios. This may be due to the fact that LEAF has fewer features compared to other simulators, as shown in Table 4. Furthermore, it is observed that the execution time for all the applications will increase with the complexity of the scenarios. However, in LEAF, the execution time of the VRgame application improves when the complexity increases (fog nodes 20) as shown in Figure 13. Considering the CPU and memory consumption we noticed fewer deviations for all applications regardless of the complexity of scenarios as shown in Figure 14 and Figure 15. As mentioned earlier, this may be due to the fact that the LEAF support only a few features and is lightweight compared to other simulators. In general, the LEAF simulator can handle all types of applications. For instance, the eHealth application performs well even when the complexity increases; however, the DCNS execution time is the worst when the complexity increases compared to other applications.

**Figure 16:**
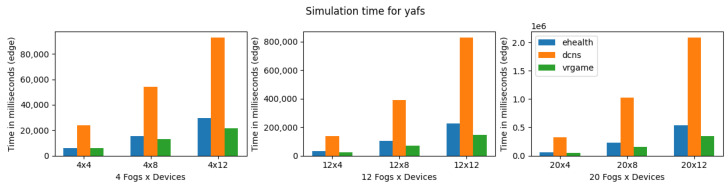
Execution time for YAFS.

**Figure 17:**
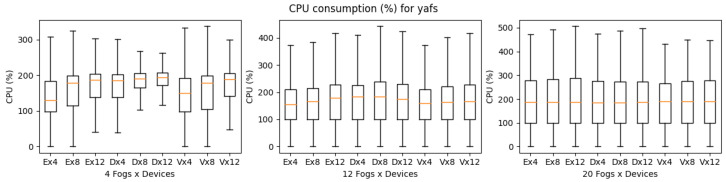
CPU consumption for YAFS during simulation (E: eHealth; D: DCNS; V: VRGame).

**Figure 18:**
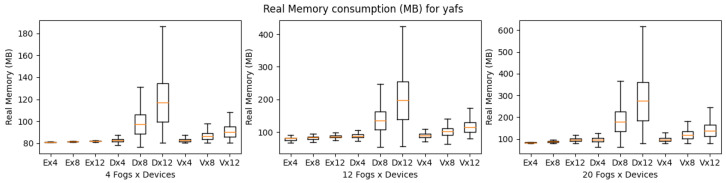
Real memory consumption for YAFS during simulation (E: eHealth; D: DCNS; V: VRGame).

YAFS provides almost the same number of features as iFogSim, as shown in Table 4. YAFS is also able to simulate all types of applications regardless of the scenario’s complexity. Considering the execution time, VRgame application executes better compared to other applications. However, in comparison with iFogSim and LEAF, the YAFS execution time is much higher as shown in Figure 16. Furthermore, it is observed that both VRgame and eHealth applications perform well compared to DCNS in terms of execution time. Considering CPU and memory consumption, DNCS consumes more resources when the complexity of the scenarios is low (fog nodes 4) as shown in Figure 17 and Figure 18. However, we observed no clear difference when the complexity of the scenarios increased (fog nodes 12 and 20).

### 4.5. Summary and Discussion

In our practical comparisons, we compared the performance of iFogSim, YAFS, and LEAF by simulating three different types of applications eHealth, DCNS, and VRGame. The practical comparison shows that YAFS performance in terms of completion time is the worst among all simulators. However, YAFS resource usage is comparatively better than iFogSim. LEAF is mainly energy consumption oriented and it performs better in terms of both execution time and resource usage. This is due to its support for fewer features when compared to iFogSim and YAFS. The simulators considered in our practical comparisons such as iFogSim, YAFS, and LEAF use different computational units (i.e., iFogSim uses MIPS and YAFS uses IPT, whereas LEAF uses imaginary units). Therefore, it is difficult to compare them side to side on the performance matrices. For this reason, each of them was discussed separately. Furthermore, there is an architectural difference between these simulators as well. For instance, by default LEAF lacks to support bi-directional communication between services. Therefore, simulating such applications with LEAF requires modification in application architecture. To conclude the performance of different applications on the simulators, it is difficult to simulate applications such as VRGame with complex scenarios in iFogSim, and the demanding application for iFogSim is the eHealth application. In addition, LEAF is much lighter compared to other simulators. Therefore, all three applications can be easily simulated on LEAF with no such big difference in performance parameters. However, for YAFS, the most demanding application we observed is eHealth and VRGame.

## 5. Open Issues and Challenges

FC technologies have advanced significantly over the last few years. These technologies play a vital role in the daily life of people. FC technologies have been applied to a variety of different areas such as smart homes, smart cities, smart grids, health care, transportation and industry 4.0 [46]. Several problems in the above-mentioned areas have been solved using fog concepts. However, fog-based solutions still require more consideration and improvements. For this reason, the researchers use simulators to validate the suitability of their solutions. Several simulation tools with different mechanisms and characteristics are available to model the FC scenarios. Each of these simulators has implementation constraints which makes them even more challenging to be adopted for some of the scenarios. Therefore, a researcher selecting a simulator for their solution faces several challenges, some of which are mentioned below.

### 5.1. Lack of Documentation

Each simulator provides a documentation manual to demonstrate its purpose and usage. It is important to note that the quality and completeness of the documentation may vary. Documentation plays an important role in maintaining the simulator by the community. Furthermore, proper documentation can help researchers to decide whether the simulator is suitable for their research or not. However, most of the available simulators lack to provide the proper explanatory documents thus making it even more difficult to understand the purpose of the simulator. Therefore, for any simulator, one of the keys is to provide proper, easy, and well-explained documentation to practitioners to efficiently utilize it.

### 5.2. Version Support

Technologies advance rapidly, therefore to cope with this rapid change the simulators need to be adapted to the new technologies. For example, some simulators developed with an older version of python should be compatible with the newer version of python. Some simulators fail to provide continuous updates of their implementations resulting in outdated versions. In addition, most of the examples developed with the older technology must be converted to newer technologies with continuous updates. Examples provided are one of the important parts of a simulator that help researchers to understand functionalities and their applicability. Therefore, giving related example scenarios covering overall functionalities for the simulators is of utmost importance as it reflects which type of scenarios can be implemented with a certain simulator. The lack of version support and fewer examples makes the selection of an appropriate simulator more challenging.

### 5.3. Support for Telco-Cloud Experiments

According to the European Telecommunications Standards Institute (ETSI) Mobile Edge Computing (MEC, 2014) standards take into account the viewpoint of the network edge, which involves deploying computational resources and network management functions (in the form of MEC servers) in close proximity to user devices (mobile base stations) [47]. In addition, NIST [48], defines FC as:

“a horizontal, physical or virtual resource paradigm that resides between smart end-devices and traditional cloud or data centers. This paradigm supports vertically-isolated, latency-sensitive applications by providing ubiquitous, scalable, layered, federated, and distributed computing, storage, and network connectivity.”

MEC and FC are complementary technologies and integrating mobile edge technologies such as 5G base stations or Radio Access Networks (RANs) into FC can enhance the performance and efficiency of mobile applications [49]. The existing simulators lack to fully support telco-cloud scenarios in 5G/6G environments. While there are some attempts in this direction such as [50], Italtel-i-Mec [51] and Simu5G [52] to support 5G and MEC-related scenarios, still, it is not easy to test complex telco-cloud scenarios (e.g., microservice scenarios expanded from the edge to the core in 5G) in a cloud-cellular environment. Therefore, support for telco-cloud scenarios in 5G/6G environments can be an important feature to address in the future.

### 5.4. Selection between Emulators, Simulators and Industry solutions

In addition to simulators, there exist some emulators for MEC scenarios such as EmuFog built on top of Max-iNet (MaxiNet is an extension of Mininet) which runs Docker-based applications on nodes connected by the simulated network [33]. AdvantEDGE is another Mobile Edge Emulation Platform (MEEP) which runs on Docker and Kubernetes [53]. Features such as orchestraters are not provided yet with these emulators [13]. Furthermore, several other emulators exist such as Fogify [54], Fogbed [55], Mockfog [56]. Therefore, the selection of an emulator or simulator is still an issue and needs to be addressed. This gets more complicated when we add the industry solutions also in the feasible candidates such as Amazon’s FreeRTOS (https://aws.amazon.com/freertos/, accessed on 10 March 2023) and iofog (https://iofog.org/, accessed on 10 March 2023).

### 5.5. Graphical Support

GUI is one of the important tools for simulators, which makes it easy for researchers to interact with the simulator’s core functionalities. However, most of these simulators either provide no graphical support or low-level graphical support. It will be interesting to see simulators where GUI allows you to configure the network, storage, and compute resources along with the strategies of resource management. In short, making easy to implement different scenarios with less or no code involved. However, for GUIs, maintaining the version support and the documentation will be a challenging task for the developers.

### 5.6. Support for Features and Consistency in Terminologies

The simulators differ in their cost, energy, network, mobility, and application models. Furthermore, some of the simulators lack to provide all of the above-mentioned models. In addition, the computational terminologies used for network, storage, and complexity vary from the simulator to simulator, e.g., for parameters such as computational unit and bandwidth, LEAF uses an imaginary unit (cu), iFogSim uses MIPS and YAFS uses IPT (instruction per tick), where 1 MIPS $=10^{6}$ IPT and 1 MIPS =1 CU. In addition, in some scenarios, details with a high granularity are not needed. Instead, these scenarios may need an abstract and high-level model both of the traffic and of the infrastructure where higher-level behavior is modeled. Therefore, the selection of a simulator is complicated and the type of scenarios and tested features also should be considered.

### 5.7. Serverless Fog Computing

Serverless computing relies on cloud providers for automatic resource management to dynamically allocate and provision resources for running an application in response to demand [57,58]. In recent years, there have been attempts to introduce serverless computing in the context of FC and edge computing paradigms, such as Fog Function [59], Edgeless [60]. However, to be able to simulate the scenarios of serverless FC, considered simulators need to focus more on resource management aspects such as resource allocation, scheduling, and scaling techniques based on the incoming user demands [61,62,63].

### 5.8. Green Fog Computing

FC’s main focus is to provide support for latency-sensitive applications [48]. However, other objectives can still be achieved along with latency in the FC paradigm. For instance, research on green FC is at a very early stage [64]. Therefore, there exist a lot of open issues in this direction, especially in mobile and wireless communication systems.

In FC paradigms, the energy consumption of distributed heterogeneous resources, as well as the price and carbon emission rate of their energy source, differ which affects carbon emission amount [65]. There are some efforts from the standardization bodies, for example, the 3rd Generation Partnership Project (3GPP) and the Institute of Electronic and Electrical Engineers (IEEEs) standardization bodies in Energy Saving (ES) standardization definition in Mobile and Wireless communication systems [66]. Therefore, energy metering and power usage predictions [15], as well as the carbon emission effect should be provided by the simulators in their future version. For instance, LEAF is one of the simulators that will focus on carbon emission in their future version as can be seen in Table 3.

## 6. Conclusions

In this work, we have discussed some of the most popular or recent simulators in FC. We compared eight recent simulators in terms of technical and non-technical properties. Furthermore, three of them were compared practically by simulating applications under different scenarios. The technical and non-technical comparison provides an overview of simulators for researchers and can be helpful for selecting the best-suited simulators for their respective research problems. Furthermore, this conceptual comparison shows that some of the simulators are built for a specific purpose and only a few continue to extend their features for solving different problems. The practical comparison helps in understanding the suitability of simulators for different types of applications. For instance, iFogSim failed to simulate VRGame application under complex scenarios. YAFS execution time is worst compared to iFogSim and LEAF. Similarly, LEAF provides better performance compared to iFogSim and YAFS. However, these conclusions are based on our current experiments without considering mobility. The conclusions may change for applications with mobility requirements or 5G/6G involvements. Therefore, in future works, we will make a deeper study on telco-cloud simulators in 5G/6G while implementing more complex scenarios with mobility and microservices. In addition, we will also consider state-of-the-art emulators for practical comparisons.

## Figures and Tables

**Table 1:** Related Work.

Ref.	Year	Citations	Environment			Comparison Criteria			Future Guidelines	Issues
			Cloud	Fog	Edge	Technical	Non-Technical	Performance		
[1]	2020	47	✓	✓		✓	*∂*	✓		Deprecated tools
[3]	2020	31	✓	✓		✓	*∂*			Limited to only analysis of cost model, Many of the issues have been resolved by the simulators in their new versions
[10]	2021	3		✓		✓	*∂*		✓	Deprecated tools and no performance comparison
[11]	2018	114	✓	✓		*∂*	*∂*	✓		Deprecated simulators and limited to a conceptual approach
[12]	2022	2	*∂*	*∂*	*∂*	*∂*				Comprehensive overview and does not provide any practical comparisons
[13]	2022	0		*∂*	✓	✓				Comprehensive overview and lacks to provide any practical comparisons
[14]	2020	15	✓	✓		✓	✓	*∂*		Most of the issues mentioned have been resolved by simulators, limited performance analysis
[15]	2020	52	✓	✓	✓	✓	✓		*∂*	Deprecated simulators, Lack of performance analysis
[16]	2020	85	✓	✓	✓	*∂*	*∂*			Limited focus of simulators, Lack of applicability details
[17]	2019	107		✓	✓	*∂*			*∂*	Deprecated simulators, Lack to provide details on most of the technical features, no performance analysis
[18]	2019	31			✓	✓			✓	Lack of Non-technical comparisons, Deprecated tools
[19]	2016	38	✓			✓				Limited to cloud simulators
[20]	2020	5		✓	✓	✓				Limited comparisons, Deprecated tools
[21]	2021	5				✓			*∂*	The considered tools mostly belongs to IoT technologies
Our Work	N/A	N/A	✓	✓	*∂*	✓	✓	✓	✓	-

Future Guidelines: Open issues that still need consideration in new or future versions of simulators. N/A: Not Applicable. ✓ denotes detail discussion. *∂* denotes partial discussion.

**Table 2:** Non-technical overview of simulators (Consulted on 15 June 2022).

Simulators	First Release	Latest Release	Stars	Citations	Paper	GitHub	Release Frequency	Response Frequency	Installation
iFogSim	2016	2016	168	1168	[29]	[29]	deprecated	low	✓
iFogSim2	2022	2022	44	8	[35]	[36]	low	high	✓
FogNetSim++	2018	2018	8	114	[11]	[38]	low	low	✓
EdgeCloudSim	2018	2020	297	364	[24]	[39]	moderate	low	✓
FogComputingSim	2019	2019	15	N/A	[40]	[41]	low	N/A	✗
PureEdgeSim	2019	2022	85	21	[42]	[42]	moderate	high	✓
YAFS	2019	2021	55	111	[22]	[43]	moderate	high	✓
LEAF	2021	2022	59	6	[23]	[44]	moderate	high	✓

**Table 3:** Functional comparisons of simulators.

Simulators	Language	Documentation	Graphical Support	Migration Support	Mobility/ Locationaware Support	Energyaware Model	Costaware Model	Microservices Support	Future Works
iFogSim *	Java	✓	✓			✓	✓	✓	N/A
iFogSim2 *	Java	✓	✓	✓	✓	✓	✓	✓	Monetary-based policies, support for distributed ledgers and federated machine learning
FogNetSim++ ***	C++		✓		✓	✓	✓		Support for virtual machine (VM) migration and interoperability
EdgeCloudSim *	Java	✓			✓		✓		Add a hand-off mechanism to decrease the task failures
FogComputingSim **	Java		✓	✓	✓	✓	✓	✓	Refining support for mobility patterns based on real datasets and other mobile communication technologies (e.g., Wi-Fi)
PureEdgeSim *	Java		✓		✓	✓			Support for the registry and the VM migrations
YAFS	Python	✓			✓	✓	✓	✓	Power-aware management policies, Controlling the computational capacity of the resources and improvements in the nomenclature.
LEAF	Python or Java	✓				✓			Time-based and location-based calculations of the carbon emissions and electricity costs

‘*’—extends CloudSim, ‘**’—extends iFogSim, ‘***’—extends OMNeT++.

**Table 4:** Overview of performance metrics used in simulators.

Metrics		Simulators							
		iFogSim	iFogSim2	FogNetSim++	EdgeCloudSim	FogComputingSim	PureEdgeSim	YAFS	LEAF
CPU consumption		✓	✓	✓	✓	✓	✓	✓	
Memory consumption		✓	✓	✓	✓	✓		✓	
Bandwidth consumption		✓	✓	✓	✓	✓	✓		
Energy consumption	Infrastructure Nodes	✓	✓	✓		✓	✓	✓	✓
	Network	✓	✓				✓		✓
	Application								✓
	Technology						✓		✓
	Carbon emissions								
Deployment cost		✓	✓		✓	✓		✓	
Latency		✓	✓	✓	✓	✓		✓	✓
Execution time		✓	✓	✓	✓	✓	✓		
CPU time		✓	✓		✓	✓		✓	
Network time		✓	✓		✓	✓		✓	
Migration time		✓	✓			✓			
Failed tasks		✓	✓		✓	✓	✓		
Waiting time				✓			✓	✓	
Link availability									
Node availability								✓	

**Table 5:** Resources by layer (Adopted from [1]).

Resources/Layer	Cloud	Fog	IoT
CPU	44,800 MIPS	2800 MIPS	1000 MIPS
Memory	40 GB	4 GB	1 GB
Bandwidth	10 GB	1 GB	100 MB

## Data Availability

All the data is available on a GitHub repository at https://github.com/gphilippee/fog-scenarios, (accessed on 10 March 2023).

## Abbreviations

The following abbreviations are used in this manuscript:

CLOUDS	Cloud computing and Distributed Systems
FC	Fog Computing
EC	Edge Computing
IoT	Internet of Things
MEC	Multi-access Edge Computing
GUI	Graphical User Interface
RANs	Radio Access Networks
